# Phosphatase and tensin homolog (PTEN) in antigen-presenting cells controls Th17-mediated autoimmune arthritis

**DOI:** 10.1186/s13075-015-0742-y

**Published:** 2015-08-26

**Authors:** Stephan Blüml, Emine Sahin, Victoria Saferding, Eliana Goncalves-Alves, Eva Hainzl, Birgit Niederreiter, Anastasia Hladik, Tobias Lohmeyer, Julia S. Brunner, Michael Bonelli, Marije I. Koenders, Wim B. van den Berg, Giulio Superti-Furga, Josef S. Smolen, Gernot Schabbauer, Kurt Redlich

**Affiliations:** Division of Rheumatology, Internal Medicine III, Medical University of Vienna, Währinger Gürtel 18-20, A-1090 Vienna, Austria; Institute for Physiology, Center for Physiology and Pharmacology, Medical University Vienna, Schwarzspanierstrasse 17, A-1090 Vienna, Austria; Rheumatology Research and Advanced Therapeutics, Department of Rheumatology, Radboud University Nijmegen Medical Center, Geert Grooteplein-Zuid 10, 6525 GA Nijmegen, The Netherlands; CeMM - Center for Molecular Medicine of the Austrian Academy of Sciences, Lazarettgasse 14, Vienna, 1090 Austria

## Abstract

**Introduction:**

Autoreactive T cells are a central element in many systemic autoimmune diseases. The generation of these pathogenic T cells is instructed by antigen-presenting cells (APCs). However, signaling pathways in APCs that drive autoimmune diseases, such as rheumatoid arthritis, are not understood.

**Methods:**

We measured phenotypic maturation, cytokine production and induction of T cell proliferation of APCs derived from wt mice and mice with a myeloid-specific deletion of PTEN (myeloid PTEN^-/-^) in vitro and in vivo. We induced collagen-induced arthritis (CIA) and K/BxN serum transfer arthritis in wt and myeloid-specific PTEN^-/-^ mice. We measured the cellular composition of lymph nodes by flow cytometry and cytokines in serum and after ex vivo stimulation of T cells.

**Results:**

We show that myeloid-specific PTEN^-/-^ mice are almost protected from CIA. Myeloid-specific deletion of PTEN leads to a significant reduction of cytokine expression pivotal for the induction of systemic autoimmunity such as interleukin (IL)-23 and IL-6, leading to a significant reduction of a Th17 type of immune response characterized by reduced production of IL-17 and IL-22. In contrast, myeloid-specific PTEN deficiency did not affect K/BxN serum transfer arthritis, which is independent of the adaptive immune system and solely depends on innate effector functions.

**Conclusions:**

These data demonstrate that the presence of PTEN in myeloid cells is required for the development of CIA. Deletion of PTEN in myeloid cells inhibits the development of autoimmune arthritis by preventing the generation of a pathogenic Th17 type of immune response.

**Electronic supplementary material:**

The online version of this article (doi:10.1186/s13075-015-0742-y) contains supplementary material, which is available to authorized users.

## Introduction

Inflammatory joint diseases, such as rheumatoid arthritis (RA) or psoriatic arthritis (PsA), are chronic disorders that affect more than 1 % of the population and lead to significant disability [1, 2]. The hallmark of RA is local bone destruction mediated by cells of the innate immune system, termed osteoclasts. However, genetic associations with major histocompatibility complex (MHC) II molecules, the presence of autoantibodies such as rheumatoid factor or anti-citrullinated peptide antibodies, as well as high amounts of T cells in the inflamed synovial membrane suggest an important involvement of the adaptive immune system [3–6]. Cumulative evidence indicates that CD4^+^ T cells, especially those polarized toward the T helper (Th) 1/Th17 subsets, play a critical role in the pathogenesis of both RA and PsA [7–9]. Not only the signature cytokine interleukin (IL)-17 produced by these Th17 cells, but also IL-21 and IL-22, have been demonstrated to be present in RA synovial membrane and fluid [10, 11]. Furthermore Th17 cells were shown to be involved in various key processes in arthritis development such as pannus formation by activation of synovial fibroblasts and joint destruction by induction of bone-resorbing osteoclasts [10–12]. As a consequence, there is a strong interest in defining the conditions and factors as well as signaling pathways determining development and activity of these pathogenic Th17 cells.

Among various factors involved in the activation of Th17 cells, antigen-presenting cells (APCs) are thought to be essential. APCs orchestrate the generation of adaptive immune responses by controlling the activation of antigen-specific T cells [13], as costimulatory molecules such as CD80 and CD86 provided by APCs are required to enable activation of naïve T cells via MHC-peptide complexes [14, 15]. In addition, APCs determine T cell polarization by the cytokine pattern they release [16, 17]. For example, IL-23, IL-6, and IL-1ß, have been shown to be indispensable for T cell polarization toward the pathogenic Th17 subset and are therefore also important for the development of various autoimmune conditions [12, 18, 19]. However, signal transduction pathways in APCs that govern the subsequent development of Th17 cells in vivo have not been identified yet.

The phosphatidylinositol 3-kinase (PI3K) pathway is one of the most important signal transduction pathways, regulating not only fundamental processes such as cell survival, cell migration, proliferation and cytoskeleton remodeling [20–22] but also leukocyte activation and immune cell homeostasis [23, 24].

Moreover, PI3K-γ but also PI3K-δ, PI3K family members enriched in leukocytes are involved in the pathogenesis of arthritis. Blocking of PI3K-γ or PI3K-δ with antibodies or their genetic deletion has been shown to diminish inflammatory arthritis, due to reduction of leukocyte migration into the inflamed joints [25–27]. However, to date, there are no data available about the contribution of the PI3K pathway in APCs in the induction of autoimmunity.

Phosphatase and tensin homolog (PTEN) is a phosphatase antagonizing all classes of PI3K [20, 28]. Using a genetic approach, where PTEN is deleted only in myeloid cells (myeloid *pten*^*-/-*^), allowed us therefore to investigate the role of the PI3K pathway in autoimmunity, specifically in APCs.

## Methods

### Antibodies and reagents

Antibodies for western blotting, immunohistochemistry and fluorescence-activated cell sorting (FACS) analysis were obtained from: CD11b (Serotec, Raleigh, NC, USA), CD11c, Gr1, CD25, CD40, CD80, CD86, I-A/I-E: all BD Biosciences (San Jose, CA, USA), CD4: Beckman Coulter, (Brea, CA, USA), FoxP3: eBioscience (San Diego, CA, USA). Lipopolysaccharide (LPS) and CpG DNA were obtained from Invivogen (San Diego, CA, USA). Wortmannin was from Sigma-Aldrich (St Louis, MO, USA). Anti-collagen enzyme-linked immunosorbent assay (ELISA), Mouse Th1/Th2 10plex FlowCytomix Multiplex as well as IL-23 and IL-22 FlowCytomix simplex were from Bender MedSystems (Vienna, Austria) and used according to the manufacturer’s protocols. Antibodies against phospho-AKT, AKT, were from Cell Signaling (Danvers, MA, USA). ELISA antibodies against murine IL-6, IL-12/23, interferon (IFN)-γ, IL-17A, IL-22, IL-4 were obtained from eBioscience.

### Mice

*Pten*^flox/flox^ mice were provided by Dr. Tak W. Mak. These mice were crossed with mice expressing the Cre recombinase under the control of the lysozyme M (LysM) promoter [29] to generate LysMCre*pten*^flox/flox^ (myeloid *pten*^-/-^) mice. All mice were on a C57BL/6 J background. For arthritis experiments, littermates of LysMCre*pten*^flox/flox^ (either Cre + (myeloid *pten*^-/-^) or Cre – (wild-type (wt)) were used.

All animal studies were approved by the animal ethics committee of the Medical University Vienna and comply with institutional guidelines (BMWF-66.009/0103-C/GT/2007 and BMWF-66.009/0241-II/3b/2011).

### Bone marrow-derived dendritic cells

Bone marrow-derived dendritic cells (BMDCs) were generated as previously described [30]. BMDCs from wt mice were cultured in the presence of 10 ng/ml granulocyte macrophage colony-stimulating factor (GM-CSF), with supplementation on days 0, 3, and 6. On day 7, they were analyzed by FACS. The BMDC purity was 65–75 % CD11c^+^ and Gr1^−^. They were stimulated with 100 ng/ml LPS or 10 μg/ml CpG.

### Western blotting

BMDC derived from wt or myeloid *pten*^*-/-*^ mice were stimulated with LPS or CpG or medium alone ranging from 5 to 120 minutes. After stimulation, dendritic cells (DCs) were lysed in Laemmli buffer, and proteins were separated by electrophoresis on 10 % SDS-polyacrylamide gels. Proteins were blotted onto polyvinylidene difluoride membrane and, after blocking with 5 % dry milk/0.1 % Tween 20, incubated with primary antibodies in the same solution. Bound antibodies were detected by anti-immunoglobulin G (IgG) conjugated with peroxidase and underwent subsequent chemiluminescent detection.

### T cell stimulation in vivo

Carboxyfluorescein succinimidyl ester (CFSE) (Molecular Probes, Eugene, OR, USA) -labeled OTII cells (3–4 × 10^6^/mouse) were transferred intravenously (i.v.). After 24 hours, mice were immunized i.v. with 300 μg/mouse OVA protein (Sigma-Aldrich) together with 75 μg/mouse LPS (Sigma-Aldrich), or 75 μg LPS alone. After 4 days, spleen cells were isolated and CFSE dilution of CD4^+^ and Vα2^+^ cells was measured by flow cytometry.

### Quantitative real-time polymerase chain reaction

Total RNA was isolated from cultivated BMDCs or osteoclasts using the RNeasy Mini kit (Qiagen, Venlo, The Netherlands). One micron of total RNA was used for first-strand cDNA synthesis (Amersham Biosciences, Little Chalfont, UK) and 1 μl cDNA will then be used for PCR with specific primers. Primers used were: IL-12p40 fwd: 5′-GAC ACG CCT GAA GAA GAT GAC-3′; IL-12p40 rev.: 5′-TAG TCC CTT TGG TCC AGT GTG-3′; IL-23p19 fwd: 5′-ATG CTG GAT TGC AGA GCA GTA-3′; IL-23p19 rev: 5′-ACG GGG CAC ATT ATT TTT AGT CT-3′; IL-12p35 fwd: 5′-CCC TTG CCC TCC TAA ACC AC-3 ′, IL-12p35 rev: 5′-AAG GAA CCC TTA GAG TGC TTA CT-3 ′, IL-17A fwd: 5′-TCT CAT CCA GCA AGA GAT CC-3′, IL-17A rev: 5′-AGT TTG GGA CCC CTT TAC AC-3′, IL-4 fwd: 5′-ACG GCA CAG AGC TAT TGA TGG-3′, IL-4 rev: 5′-CGA TGA ATC CAG GCA TCG AA-3′, IFN-γ fwd: 5′-GAT GCA TTC ATG AGT ATT GCC AAG T-3′, IFN-γ rev: 5′-GTG GAC CAC TCG GAT GAG CT, PTEN fwd: 5′-ACA CCG CCA AAT TTA ACT GC-3′, PTEN rev: 5′-TAC ACC AGT CCG TCC CTT TC-3′.

### Collagen-induced arthritis

Mice were immunized subcutaneously (s.c.) with 50 μg chicken collagen type II (CII) (Sigma-Aldrich) in 50 μl H_2_O emulsified in 50 μl complete Freund’s adjuvant (CFA) that was enriched with 10 μg/ml *Mycobacterium tuberculosis* (H37Ra; Difco/BD Biosciences) and scoring was performed as described [31]. Briefly, mice were evaluated weekly for symptoms of arthritis using a semiquantitative scoring system, which includes degree of joint swelling and grip strength. Briefly, joint swelling was examined using a clinical score graded from 0 to 3 (0, no swelling; 1, mild swelling of toes and ankle; 2, moderate swelling of toes and ankle 3, severe swelling of toes and ankle). In addition, grip strength of each paw was analyzed by semiquantitatively evaluating the force needed to detach the paw from a wire 3 mm in diameter using a score from 0 to –3 (0, normal grip strength; –1 mildly reduced grip strength; –2 moderately reduced grip strength; –3, severely reduced grip strength). Assessments were performed in a blinded fashion. Scoring was performed by the same assessor for the whole experiment.

### Evaluation of inflammation, local bone erosions and cartilage destruction by histological examination

Stainings were performed as previously described [32]. For exact quantification of the areas of inflammation, hematoxylin and eosin (H&E) sections were evaluated using Osteomeasure software (OsteoMetrics, Atlanta, GA, USA).

### Serum transfer arthritis

After intraperitoneal (i.p.) application of 150 μl of K/BxN serum at day 1 and day 3, mice were scored clinically every other day with a semiquantitative scoring system as for collagen-induced arthritis (CIA) (see above) and sacrificed at day 12 and tissue was prepared for histology.

### Flow cytometric analysis of the draining lymph nodes

Draining lymph nodes (LNs) were harvested and passed through a nylon mesh to obtain single-cell suspensions. Cells were then stained with the indicated antibodies and analyzed by flow cytometry (BD FACScanto II, FACSdiva software) (BD Biosciences).

## Results

### PTEN regulates T cell-polarizing cytokines in APCs

**Fig. 1:**
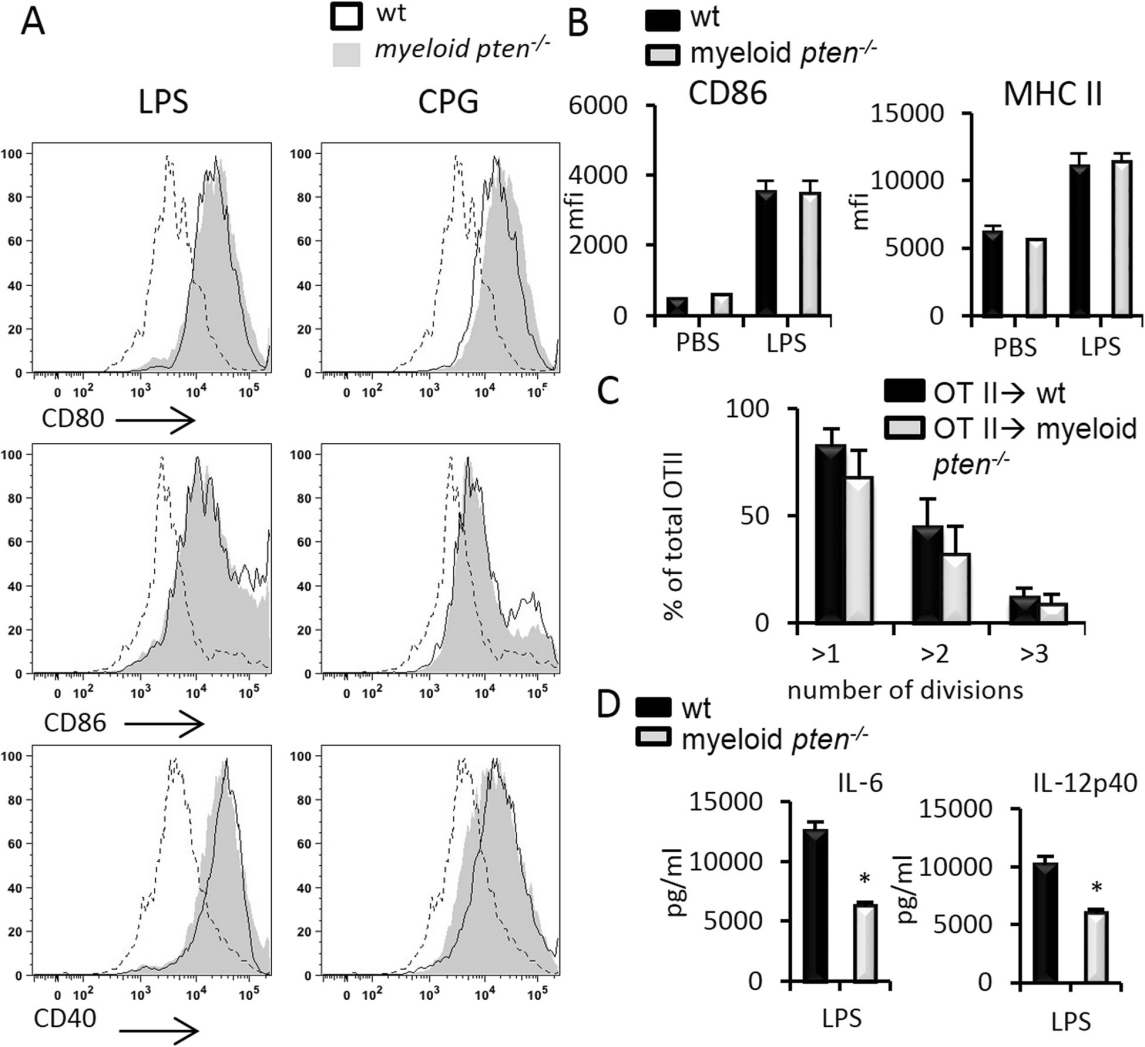
PTEN does not influence DC maturation but importantly regulates T cell polarizing cytokines in vitro*.* **a** BMDCs were either unstimulated or stimulated with the TLR ligands LPS (100 ng/ml) or CpG (5 μg/ml) and analyzed by flow cytometry. Plots show indicated surface markers on gated CD11c^+^ cells. Data are representative of at least three experiments. **b** Upregulation of CD86 and MHCII on CD11c^+^ cells in wt (n = 5) and myeloid *pten* ^-/-^ (n = 5) after administration of LPS (75 μg) i.p. measured by flow cytometry. **c** Analysis of CFSE dilution of transferred OTII cells 3 days after transfer into wt or myeloid *pten* ^-/-^ mice and immunization with LPS and ovalbumin i.v. **d** Analysis of levels of IL-6 and IL-12p40 in the supernatants of wt or myeloid *pten* ^-/-^ BMDCs stimulated with LPS. *BMDC* bone marrow-derived dendritic cell, *CFSE* carboxyfluorescein succinimidyl ester, *DC* dendritic cell, *IL* interleukin, i.p. intraperitoneally, i.v. intravenously, *LPS* lipopolysaccharide, *MHC* major histocompatibility complex, *PTEN* phosphatase and tensin homolog, *TLR* Toll-like receptor, *wt* wild-type

We first stimulated bone marrow cells from wt and myeloid *pten*^*-/-*^ animals with GM-CSF to generate bone marrow-derived DCs (BMDCs). We confirmed by quantitative real-time polymerase chain reaction (qPCR) that BMDCs from myeloid *pten*^*-/-*^ were indeed deficient in PTEN (Additional file 1). We then stimulated these cells with LPS, a ligand for Toll-like receptor (TLR) 4, and CpG DNA, a ligand for TLR9, to induce phenotypic maturation in vitro. We found no differences regarding the upregulation of costimulatory molecules such as CD80, CD86 or CD40 between *pten*^-/-^ and wt BMDCs (Fig. 1a).

To investigate maturation of primary DCs in vivo, we analyzed splenic DCs of wt and myeloid *pten*^*-/-*^ animals after i.p. challenge with LPS. In line with the data obtained in vitro, we did not detect differences in the upregulation of CD86 or MHC II on CD11c^+^ cells (Fig. 1b).

In order to test the ability of APCs from wt or myeloid *pten*^*-/-*^ animals to induce proliferation of T cells, we transferred CFSE-labeled OTII cells into wt and myeloid *pten*^*-/-*^ animals and measured proliferation in the OTII cell transplant after immunization with ovalbumin plus LPS by CFSE dilution ex vivo. We did not detect differences in the proliferation of OTII cells transferred into wt or myeloid *pten*^*-/-*^ animals after immunization (Fig. 1c).

Taken together, these data show that neither APC maturation nor primary T cell activation was altered in myeloid *pten*^*-/-*^ animals when compared to wt animals.

We next analyzed the production of IL-6 and IL-12p40 in BMDCs after LPS challenge. Induction of both cytokines was significantly lower in myeloid *pten*^-/-^ BMDCs compared to littermate-derived wt cells. Looking at RNA levels, we also found reduced upregulation of the IL-23 and IL-12 subunits IL-23p19, IL-12p40 and IL-12p35, IL-6 as well as IFN-β in myeloid *pten*^-/-^ BMDCs compared to wt cells (Fig. 1d and Additional file 1 B, C). The reduced expression of IL-12p40 and IL-23p19 in BMDCs derived from myeloid pten^-/-^ mice could be reversed by the addition of the PI3K inhibitor wortmannin, indicating a PI3K-dependent effect (Additional file 2A). Western blot analysis confirmed increased phosphorylation of AKT under basal and LPS-stimulated conditions (Additional file 2B). Taken together, these data reveal that PTEN deficiency in BMDCs leads to constitutive activation of the PI3K pathway with reduced production of polarizing cytokines albeit an intact capacity to activate naïve T cells.

### PTEN deficiency in APCs prevents collagen-induced arthritis

**Fig. 2:**
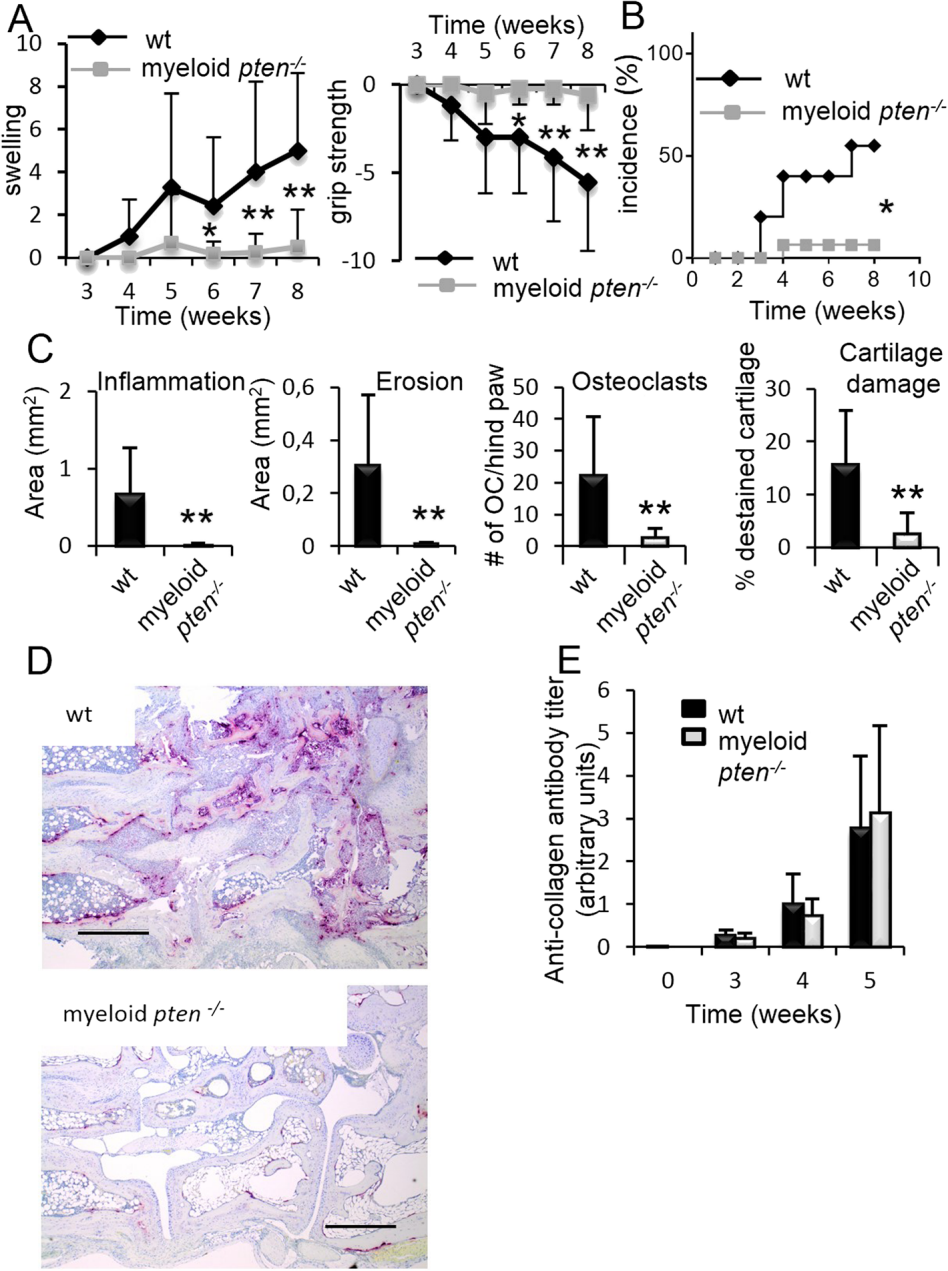
Myeloid *pten* ^-/-^ animals are protected from collagen-induced arthritis (CIA). **a** Clinical quantification of paw swelling in wt (n = 7) and myeloid *pten* ^-/-^ (n = 13) mice after induction of CIA. Data are expressed as means ± s.e. *P ≤ 0.05, **P ≤ 0.01. **b** Incidence plot of arthritis of wt and myeloid *pten* ^-/-^ after induction of CIA (^*^ *P* ≤ 0.05). Plot shows data from one representative experiment of two with similar results. **c** Quantitative histomorphometric analysis of hind paws of wt and myeloid *pten* ^-/-^ mice after induction of CIA. Data are expressed as means ± s.d. *P ≤ 0.05, **P ≤ 0.01. **d** Representative images and TRAP-stained histologies of hind paws of wt and myeloid *pten* ^-/-^ mice. Scale bars, 0.5 mm. **e** Quantification of anti-collagen IgG antibody levels of wt (n = 7) and myeloid *pten* ^-/-^ (n = 13) mice after induction of CIA. Data are means ± s.d. *IgG* immunoglobulin G, *TRAP* tartrate-resistant acid phosphatase, *wt* wild-type

We next investigated the influence of myeloid PTEN deficiency in CIA, a well-established animal model of human RA. We found that myeloid *pten*^*-/-*^ animals developed hardly any clinical symptoms of arthritis (Fig. 2a). In addition, the incidence of arthritis was significantly lower in myeloid *pten*^*-/-*^ animals (Fig. 2b). In line with this, we detected almost no synovial inflammation or local bone destruction (erosions) in histological sections of myeloid *pten*^*-/-*^ compared to wt animals (Fig. 2c). In addition, we found significantly reduced numbers of osteoclasts as well as the amount of cartilage damage in the joints of myeloid *pten*^*-/-*^ animals compared to wt animals (Fig. 2c and d). Interestingly, we detected no difference in the anti-collagen IgG titers, excluding the possibility that reduced production of anti-collagen antibodies prevented the development of arthritis in myeloid *pten*^*-/-*^ animals (Fig. 2e).

**Fig. 3:**
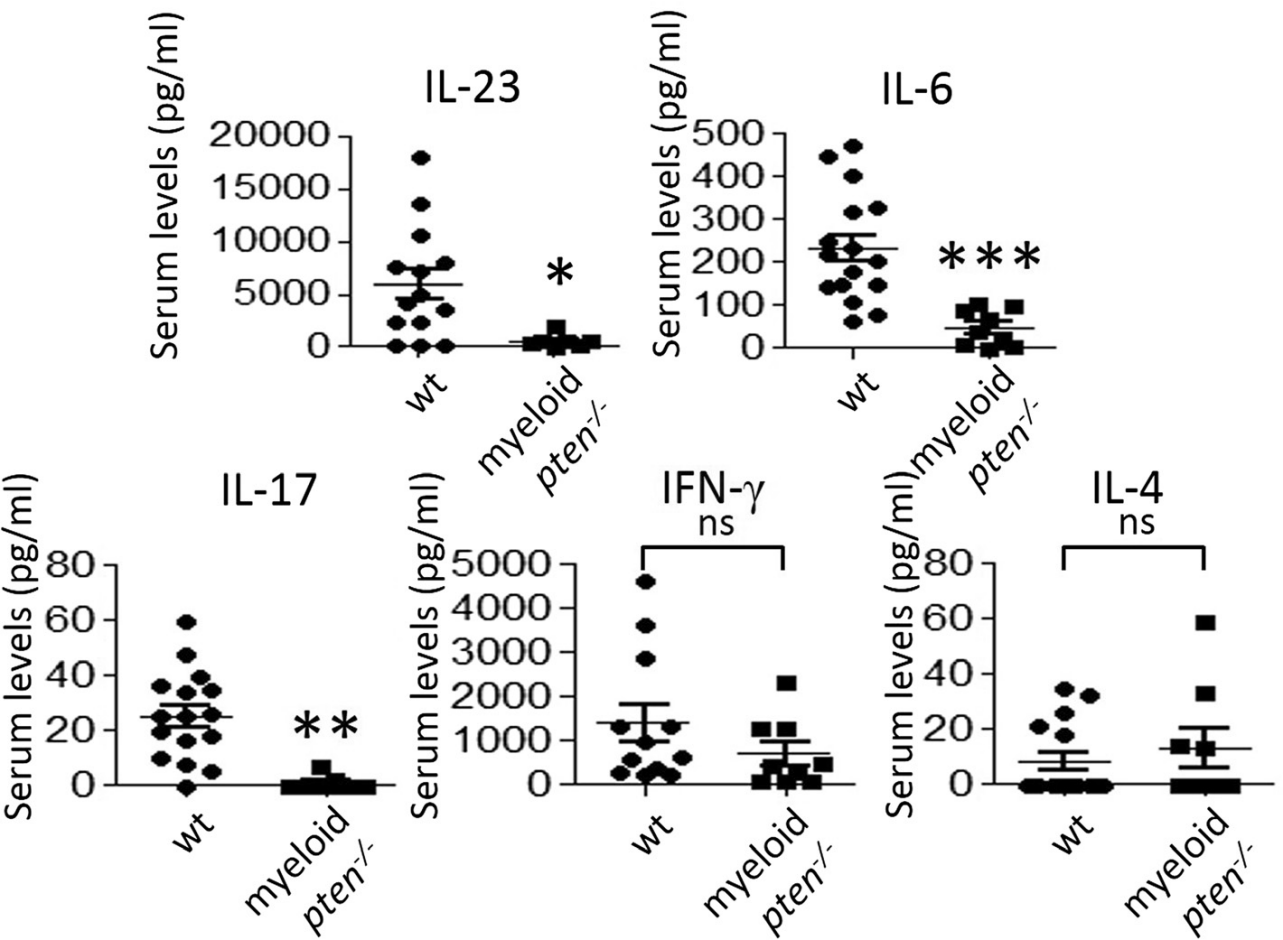
Diminished induction of Th17-related cytokines in myeloid *pten* ^-/-^ mice after induction of collagen-induced arthritis (CIA). Serum samples of wt (n = 16) and myeloid *pten* ^-/-^ (n = 9) mice obtained 4 weeks after induction of CIA. Levels of the indicated cytokines were analyzed by ELISA. Horizontal bars are means of each group ± s.e. Each data point represents an individual animal *P ≤ 0.05, **P ≤ 0.01, ^***^ *P* ≤ 0.001. *ELISA* enzyme-linked immunosorbent assay, *Th* T helper, *wt* wild-type

In order to understand the mechanism of the protective effect of myeloid *pten*^*-/-*^ animals in CIA, we first analyzed serum levels of proinflammatory cytokines 4 weeks after induction of CIA. We measured profoundly reduced amounts of the Th17-related cytokines IL-17 and IL-23 as well as IL-6 in myeloid *pten*^*-/-*^ mice compared to wt littermates. Serum levels of interferon-γ and IL-4, however, were not different between the two groups (Fig. 3).

**Fig. 4:**
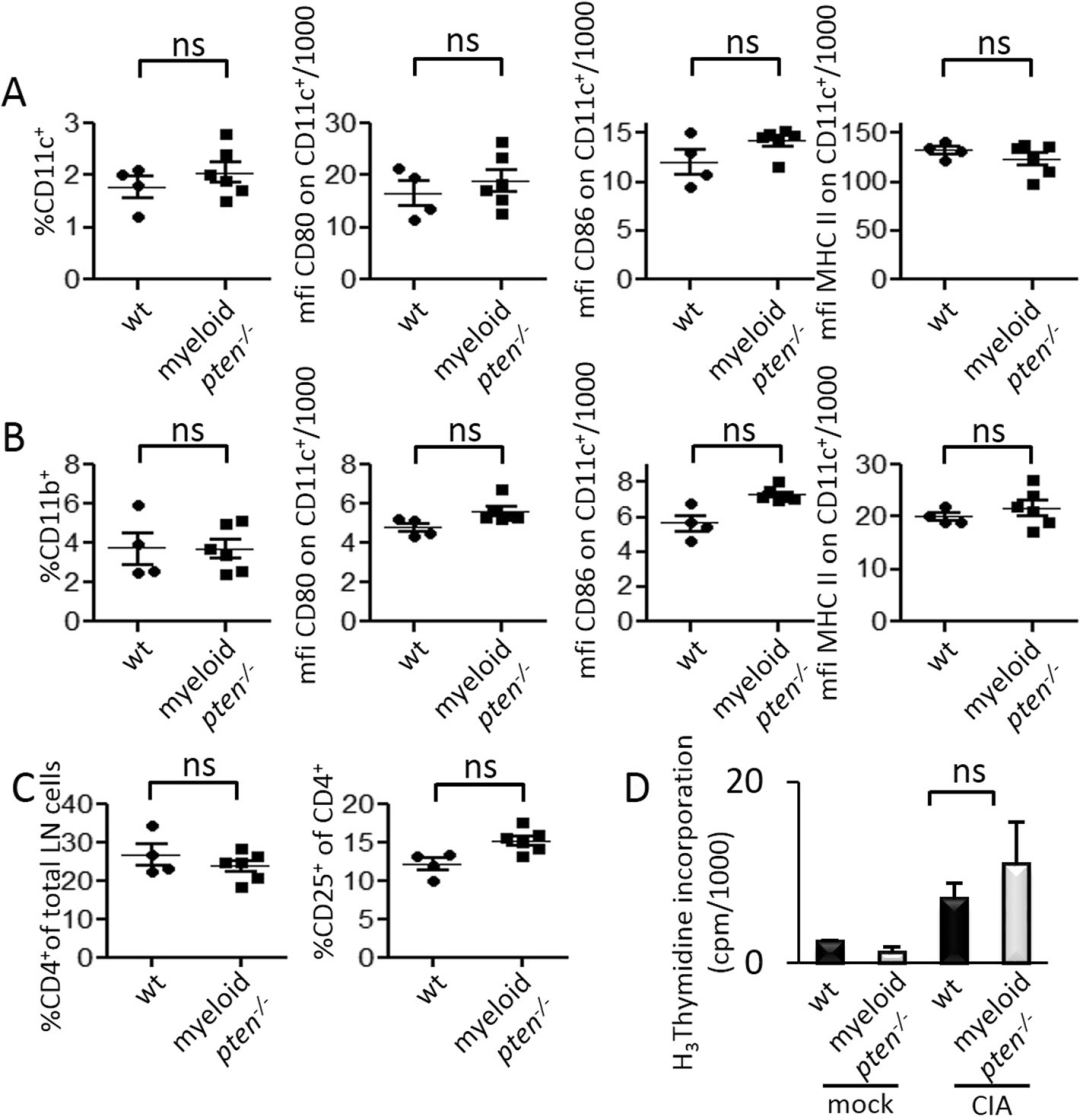
PTEN is not critically involved in APC migration and phenotypic maturation in vivo. **a**–**c** Cells of draining LN from wt (n = 4) and myeloid pten ^-/-^ mice (n = 6) 2 weeks after induction of CIA and analyzed by flow cytometry. CD11c^+^ DCs (**a**) and CD11b^+^ macrophages (**b**) (shown as percent of total LN cells), and expression levels of CD80, CD86 and MHC II (mean fluorescence intensity, mfi) on CD11c^+^ cells or CD11b^+^ cells. **c** CD4^+^ T cells and CD4^+^CD25^+^ activated T cells (shown as percent of total CD4^+^ cells). *Horizontal bars* are means of each group ± s.e. **d** Spleen cells from wt or myeloid pten ^-/-^ mice of mock immunized (adjuvants only, *left two bars*) or after immunization with collagen (*right two bars*) were restimulated with 100 μg/ml collagen II in vitro and proliferation was quantified by H_3_Thymidine incorporation. Data are expressed as means ± s.d. *APC* antigen-presenting cell, *CIA* collagen-induced arthritis, *DCs* dendritic cells, *LN* lymph node, *MHC* major histocompatibility complex, *PTEN* phosphatase and tensin homolog

We next evaluated, whether PTEN deficiency in myeloid cells influences the induction of an antigen-specific cellular immune response to collagen. As migration of antigen-loaded APCs to the draining LN is a prerequisite for this response in vivo, we first analyzed the cellular composition of draining LNs in wt and myeloid *pten*^*-/-*^ animals after induction of CIA. We found no difference between myeloid *pten*^*-/-*^ and wt animals in the relative abundance of APCs, such as CD11c^+^ DCs or CD11b^+^ macrophages (Fig. 4a and b). We next analyzed the maturation state of APCs in the draining LN in vivo and found, in line with our in vitro data, no significant differences in the surface expression of CD80, CD86 or MHC II on CD11c^+^ or CD11b^+^ cells (Fig. 4a and b).

Of note, the relative numbers of CD4^+^ T cells as well as activated (CD4^+^CD25^+^) T cells did not differ between the groups (Fig. 4c). Moreover, in vitro restimulation of spleen cells with collagen 14 days after induction of CIA resulted in similar proliferation of T cells in wt and myeloid *pten*^*-/-*^ mice (Fig. 4d), suggesting that antigen-specific T cell activation after induction of CIA is not altered by the absence of PTEN in myeloid cells.

These data demonstrate that the marked reduction of joint inflammation in myeloid *pten*^*-/-*^ mice is not caused by alterations of the migratory pattern, the maturation state of APCs or the subsequent T cell activation after immunization with collagen.

### PTEN in APCs allows for priming of Th17 cells in vivo

**Fig. 5:**
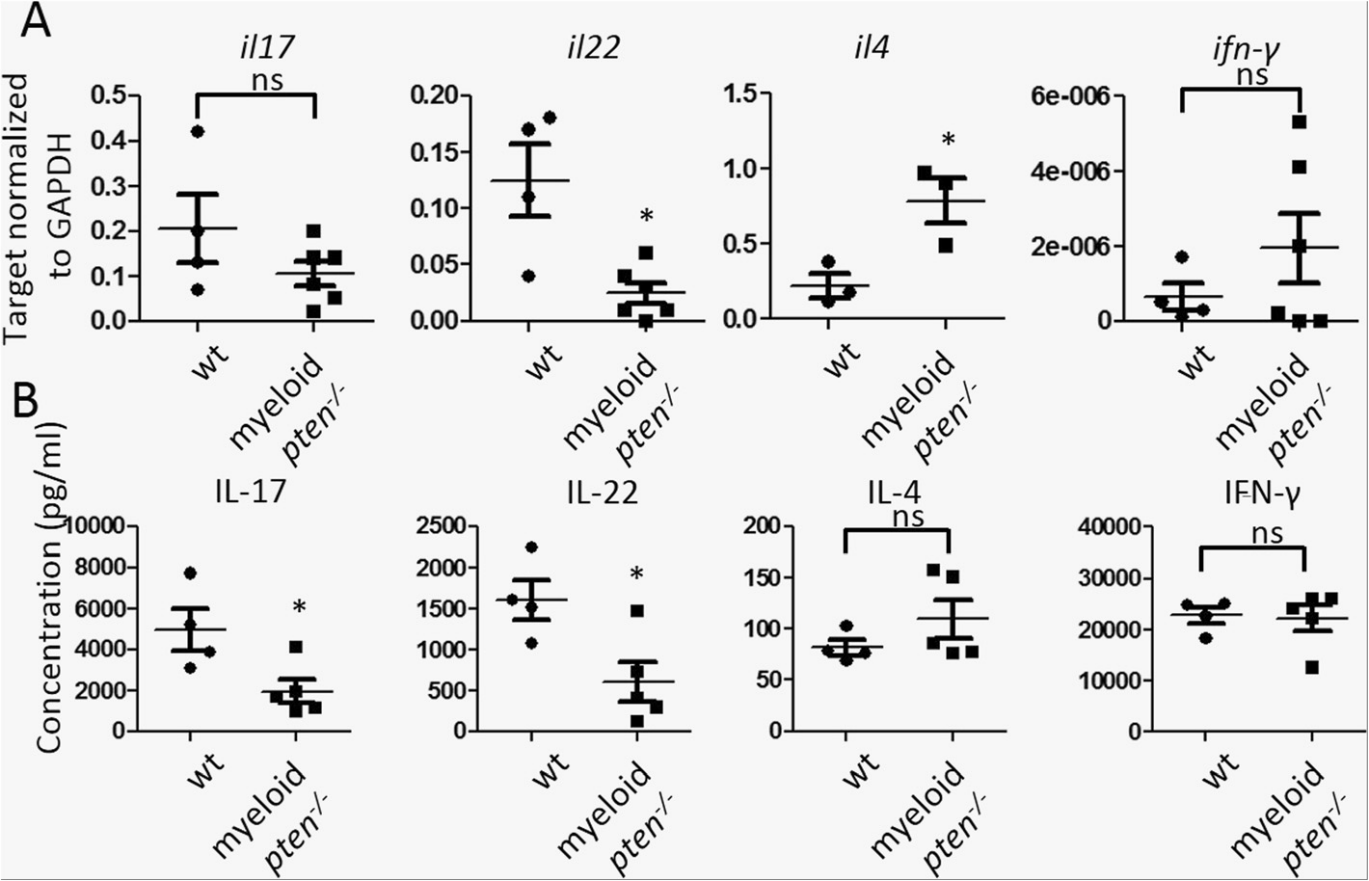
PTEN in APCs allows for priming of Th17 cells in vivo. **a**, **b** Cells of draining LN of wt (n = 4) and myeloid *pten* ^-/-^ (n = 6) mice were harvested 2 weeks after induction of CIA. **a** mRNA levels of the indicated cytokines were determined by qPCR. *Horizontal bars* are means of each group ± s.e. Each data point represents an individual animal *P ≤ 0.05. **b** LN cells of wt and myeloid *pten* ^-/-^ mice were stimulated in vitro with anti-CD3 for 3 days and levels of the indicated cytokines in the supernatant were measured by ELISA. *Horizontal bars* are means of each group ± s.d. Each data point represents an individual animal *P ≤ 0.05. *APCs* antigen-presenting cells, *CIA* collagen-induced arthritis, *ELISA* enzyme-linked immunosorbent assay, *LN* lymph node, *PTEN* phosphatase and tensin homolog, *qPCR* quantitative real-time polymerase chain reaction, *Th* T helper, *wt* wild-type

As the development of CIA critically depends on the generation of Th17 cells [12, 33] and we have found reduced levels of Th17-related cytokines in the serum of myeloid *pten*^*-/-*^ mice, we next analyzed the impact of PTEN deficiency in myeloid cells on T cell polarization in CIA. Therefore, we harvested the draining LNs of wt and myeloid *pten*^*-/-*^ mice 14 days after immunization with collagen and determined mRNA expression of cytokines specific for polarized T cells by qPCR. We found a reduction of the mRNA transcripts specific for Th17 cytokines, i.e., IL-17 and IL-22, and concomitantly an increase of the Th2 cytokine IL-4, whereas mRNA levels of the Th1 cytokine IFN-γ were not altered between the groups (Fig. 5a). Analysis of supernatants from draining LN cells after in vitro stimulation with anti-CD3 revealed a similar pattern. We detected a significant reduction of the Th17 cytokines IL-17 as well as IL-22. There was a trend toward higher IL-4 levels, while IFN-γ expression was unchanged (Fig. 5b). The proliferative response after anti-CD3 stimulation was equivalent in LN cells of both groups (Additional file 3).

These data demonstrate reduced priming of Th17 cells in myeloid *pten*^*-/-*^ mice, with concomitant slight increase in the production of Th2 cytokines. Hence, myeloid *pten*^*-/-*^ mice are protected from CIA due to reduced Th17 priming capabilities of PTEN^-/-^ APCs. These data also confirm the essential role of the PI3K/PTEN pathway in myeloid cells in the generation of Th17-mediated autoimmune disease.

### Absence of PTEN in APCs does not affect the development of arthritis that is solely dependent on innate effector mechanisms

In myeloid *pten*^*-/-*^ mice not only APCs but also neutrophil granulocytes and macrophages are lacking PTEN. Consequently we tested whether the absence of PTEN in granulocytes and macrophages might reduce their potential to migrate into the synovial membrane or their destructive capabilities using the K/BXN serum transfer arthritis model [34, 35]. This model depends only on innate effector mechanisms, as a cocktail of arthritogenic antibodies transferred into wt recipients is sufficient to induce an inflammatory erosive arthritis even in the absence of a functioning adaptive immune system [35, 36].

**Fig. 6:**
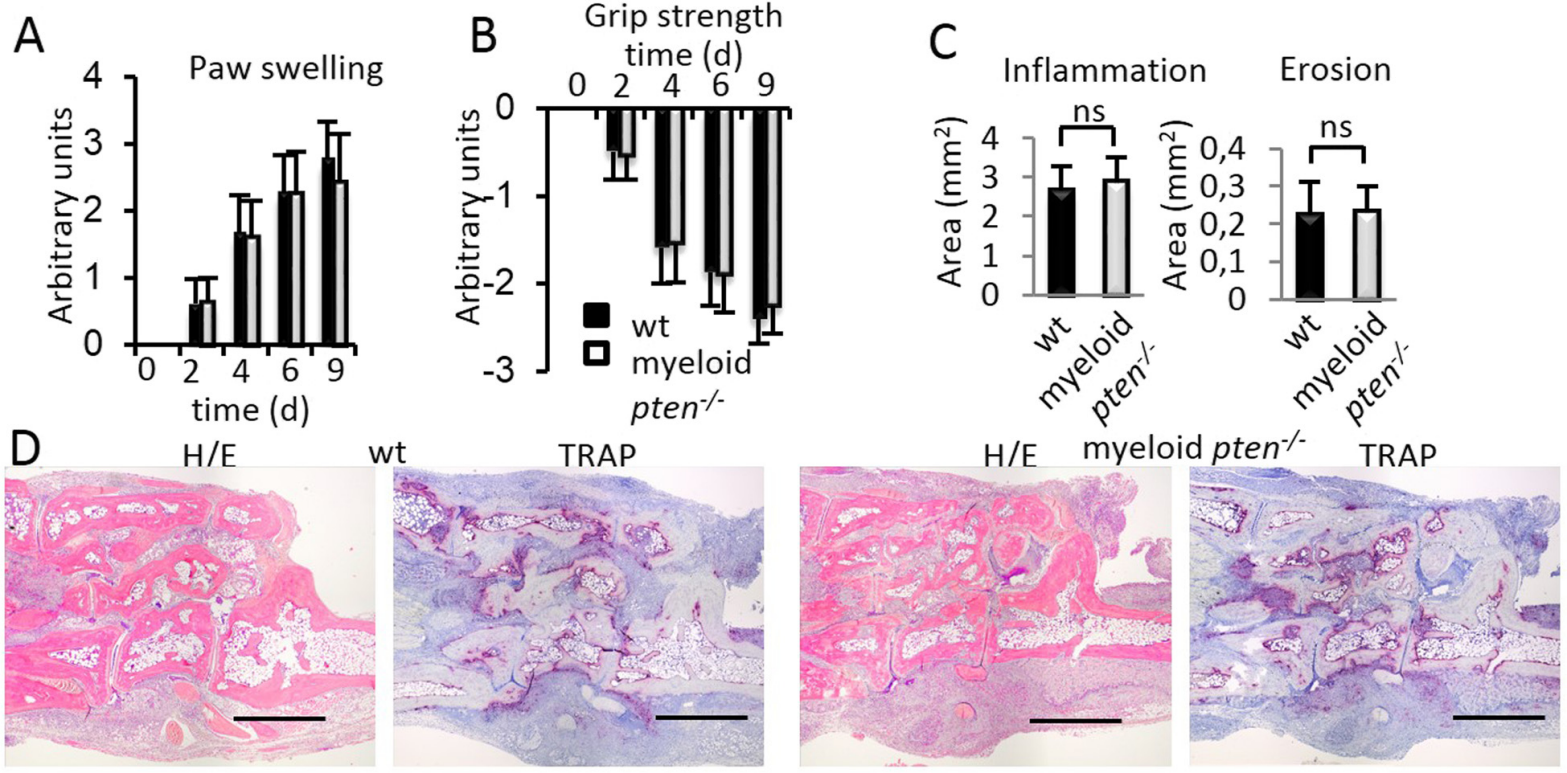
Absence of PTEN in APCs does not affect the development of innate immunity-dependent arthritis. **a**, **b** Clinical assessment of (**a**) paw swelling and (**b**) grip strength in wt (n = 7) and myeloid *pten* ^-/-^ (n = 7) after induction of K/BxN serum transfer arthritis. Data are expressed as means ± s.d. **C** Quantitative histomorphometric analysis of the hind paws of wt and myeloid *pten* ^-/-^ mice 12 days after induction of K/BxN serum transfer arthritis. Data are expressed as means ± s.d. *P ≤ 0.05. **d** Representative histologies of hind paws of wt and myeloid *pten* ^-/-^ mice obtained 12 days after induction of K/BxN serum transfer arthritis (H&E staining, TRAP staining). Scale bars, 1 mm. *APCs* antigen-presenting cells, *H&E* hematoxylin and eosin, *PTEN* phosphatase and tensin homolog, *TRAP* tartrate-resistant acid phosphatase, *wt* wild-type

We found, that time of onset and clinical severity of serum transfer arthritis was not altered in myeloid *pten*^*-/-*^ mice as compared to their wt littermates (Fig. 6a and b). In line, when we evaluated the histological signs of arthritis we found no difference in the degree of synovial inflammation or local bone erosions in both groups (Fig. 6c and d). These results show that effector mechanisms leading to inflammatory arthritis remain intact in myeloid *pten*^*-/-*^ animals in K/BXN serum transfer arthritis, which is independent of antigen presentation by APCs and the induction of an adaptive immune response.

## Discussion

Here we describe a novel function of the PI3K pathway in controlling autoimmune arthritis. We used conditional knockout animal models where PTEN deficiency is achieved selectively in the entire myeloid cell compartment [29, 37] with all other cell types being sufficient for PTEN. With this strategy we were able to investigate the role of PTEN in the induction of autoimmunity without any interference of potential effects of the PI3K/PTEN axis on T and B cells or the mesenchymal cell compartment. We demonstrate that PTEN deficiency in APCs almost completely prevents CIA by reducing the generation of pathogenic T helper cells. The PI3K pathway has been implicated in APCs in the regulation of T helper cell differentiation: deletion of the p85 regulatory subunit of PI3K resulted in an increased production of IL-12 in vitro, leading to enhanced Th1-mediated immunity in a Leishmania infection model [38]. In line with these data, PTEN^-/-^ APCs exhibit a reduced capacity to produce cytokines such as IL-23 and IL-6, which importantly shape subsequent Th responses [7, 17]. In our murine model for RA, this translates into a severely reduced generation of pathogenic IL-17- and IL-22-producing cells.

In CIA, significantly reduced levels of IL-17 were present in the serum and also in draining LNs after induction of CIA. However, the proliferative response of T cells stimulated by wt or PTEN-deficient DCs was comparable in vitro as well as in vivo in various analyses, demonstrating clearly that the *quality* but not the *quantity* of the T cell response was altered in the absence of PTEN in DCs. Of note, the humoral response upon immunization with collagen was comparable in wt and myeloid *pten*^*-/-*^ mice. This shows again that it is not the general activation of the adaptive immune system that is impaired in myeloid *pten*^*-/-*^ mice, but the quality of the response. In addition, we found no difference in the extent of serum transfer arthritis and we recently reported that tumor necrosis factor (TNF)-driven arthritis is even augmented in myeloid *pten*^*-/-*^ compared to wt animals, especially with respect to bone destruction, due to increased osteoclastogenic potential of PTEN-deficient myeloid cells [39]. Therefore, the major phenotype we detect in myeloid *pten*^*-/-*^ mice reflects an inability to mount an adaptive immune response that is able to induce joint pathology in these mice. Reduced production of T cell-polarizing cytokines such as IL-6 and IL-23 by myeloid *pten*^*-/-*^ APCs in vitro as well as in vivo after induction of CIA leads to reduced generation of IL-17- and IL-22-producing T cells, which in turn are required to induce arthritis in CIA animals. In contrast, in serum transfer arthritis, where the pathogenesis of joint pathology solely depends on innate effector mechanisms, we did not detect differences in clinical as well as histological signs of arthritis. Thus, in two arthritis models, which are independent of adaptive immunity, intact effector mechanisms in myeloid *pten*^*-/-*^ mice have been observed, highlighting the profound effect of PTEN in APC for the induction phase of immune-mediated arthritis.

Interestingly, activation of the PI3K pathway in CD4^+^ T cells is necessary for Th17 polarization, as demonstrated by significantly reduced generation of IL-17-producing T cells in p85α-deficient CD4 cells [40]. This suggests opposite requirements and functions of PI3-kinase signaling in APCs and T cells in Th17 polarization in vivo.

While the data presented here suggest a pivotal role of PTEN expression in APCs in the generation of autoimmune arthritis, it is conceivable that PTEN could control arthritis also via activity in other cell types. In this respect, reports demonstrating a protective role of PI3K-γ deficiency in CIA, mainly due to effects on migration of effector cells into the inflamed joints should be mentioned [25]. In addition, loss of PTEN expression in fibroblasts was noted at areas of joint damage in RA patients [41]. Furthermore, CIA was ameliorated by delivering an adenoviral construct silencing PTEN directly to the inflamed joint, suggesting events downstream of antigen presentation [42]. These observations suggest that the PI3K/PTEN axis controls multiple aspects in the pathogenesis of inflammatory diseases in different tissues such as cell migration, invasive behavior, cytokine production and proliferation, and T cell polarization. There are known examples of different effects of one and the same signal transduction pathway depending on the cell type analyzed. Rapamycin, a widely used immunosuppressive agent, has potent and well-described inhibitory effects on T cells by blocking the mechanistic target of rapamycin (mTOR) in these cells [43]. Rapamycin, on the other hand has also immunostimulatory effects on myeloid cells, which are thought to be responsible for some of its serious clinical side effects such as fever, pneumonitis or glomerulonephritis [44]. It is therefore of tremendous importance to dissect the biological effects of signal transduction pathways in a tissue-specific manner, especially in the light of possible therapeutic interventions in humans. APCs are pivotal to generate the desired immunity against microbial infections but can also initiate self-destructing autoimmunity. It is therefore imperative to decipher the molecular mechanisms controlling the immunomodulatory potential of APCs in order to enable their modification for therapeutic interventions.

## Conclusions

Our data provide the first evidence for the pivotal and previously unanticipated role of the PI3K/PTEN pathway in APCs in controlling autoimmune arthritis. Sustained PI3K signaling in myeloid cells protects from pathology via inhibition of the polarization of self-reactive Th17 cells (Additional file 4). These findings may pave the way for selective novel targeted therapies for chronic destructive arthritis and possibly other inflammatory disorders.

## Additional files

Additional file 1:
**(A) BMDCs from wt or myeloid** ***pten*** ^**-/-**^ **animals were analyzed for** ***pten*** **mRNA expression by qPCR.** (B) Quantitative PCR analysis of wt or myeloid *pten* ^-/-^ BMDCs stimulated with CpG (5 μg/ml) for the indicated mRNAs. Data are expressed as mean values ± s.d. ^*^ *P* ≤ 0.05. (JPEG 336 kb) Additional file 2:
**(A) BMDCs from wt or DC** ***pten*** ^**-/-**^ **animals were analyzed for the expression of the indicated mRNAs by qPCR.** (B) BMDCs from wt or DC *pten* ^-/-^ animals were stimulated as indicated and analyzed by western blot for the indicated proteins. (JPEG 214 kb) Additional file 3:
**Cells of the draining LN of wt (n = 4) and myeloid** ***pten*** ^**-/-**^ **(n = 6) mice 2 weeks after induction of CIA were stimulated with plate-bound anti-CD3 for 3 days.** H_3_ Thymidine incorporation was used to quantify proliferation. Data are expressed as means ± s.d. (JPEG 134 kb) Additional file 4:
**Schematic representation of APC activation in wt and myeloid** ***pten*** ^**-/-**^ **animals.** In wt, APCs are activated and initiate autoimmunity by activating and polarizing T cells toward the Th17 lineage. PTEN-deficient APCs fail to induce autoimmunity due to sustained PI3K signaling, leading to altered signaling and reduced polarizing cytokine expression. (JPEG 294 kb)

## Abbreviations

APC: antigen-presenting cell
BMDC: bone marrow-derived dendritic cell
CFA: complete Freund’s adjuvant
CFSE: carboxyfluorescein succinimidyl ester
CIA: collagen-induced arthritis
CII: type II collagen
DC: dendritic cell
ELISA: enzyme-linked immunosorbent assay
FACS: fluorescence-activated cell sorting
GM-CSF: granulocyte macrophage colony-stimulating factor
H&E: hematoxylin and eosin
i.p.: intraperitoneally
i.v.: intravenously
IFN-γ: interferon gamma
IgG: immunoglobulin G
IL: interleukin
LN: lymph node
LPS: lipopolysaccharide
LysM: lysozyme M
MHC: major histocompatibility complex
mTOR: mechanistic target of rapamycin
NS: not significant
PI3K: phosphoinositide 3-kinase
PsA: psoriatic arthritis
PTEN: phosphatase and tensin homolog
qPCR: quantitative real-time polymerase chain reaction
RA: rheumatoid arthritis
s.c.: subcutaneously
Th: T helper
TLR: Toll-like receptor
TNF: tumor necrosis factor
TRAP: tartrate-resistant acid phosphatase
wt: wild-type
