# Supplementary figures and images for: Phosphatase and tensin homolog (PTEN) in antigen-presenting cells controls Th17-mediated autoimmune arthritis
Source: Arthritis Res Ther. 2015 Aug 26;17(1):230. doi: 10.1186/s13075-015-0742-y (PMC4549861; doi:10.1186/s13075-015-0742-y)

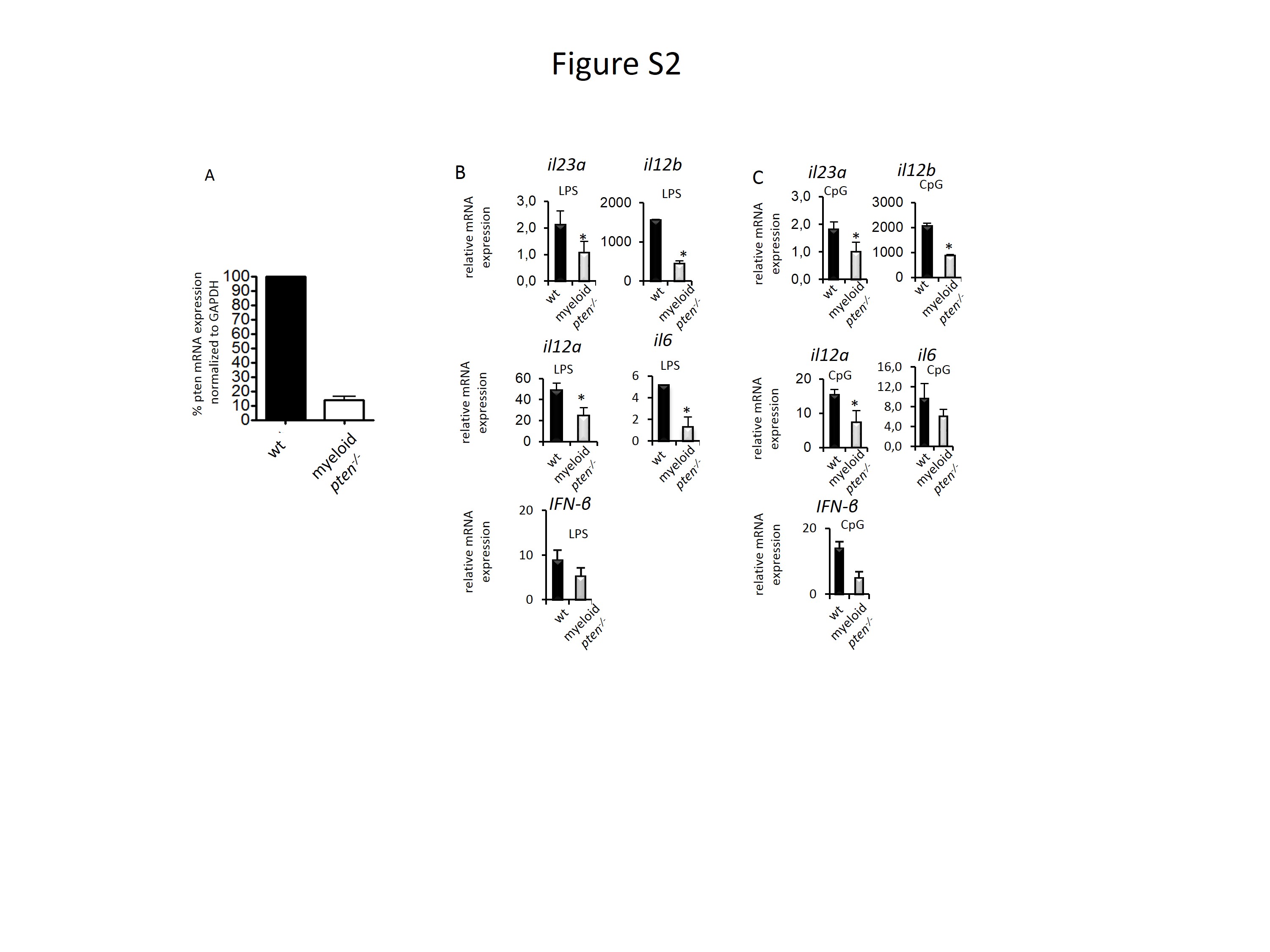

Supplement: Additional file 1: — (A) BMDCs from wt or myeloid pten -/- animals were analyzed for pten mRNA expression by qPCR. (B) Quantitative PCR analysis of wt or myeloid pten -/- BMDCs stimulated with CpG (5 μg/ml) for the indicated mRNAs. Data are expressed as mean values ± s.d. * P ≤ 0.05. (JPEG 336 kb) [file 13075_2015_742_MOESM1_ESM.jpeg]

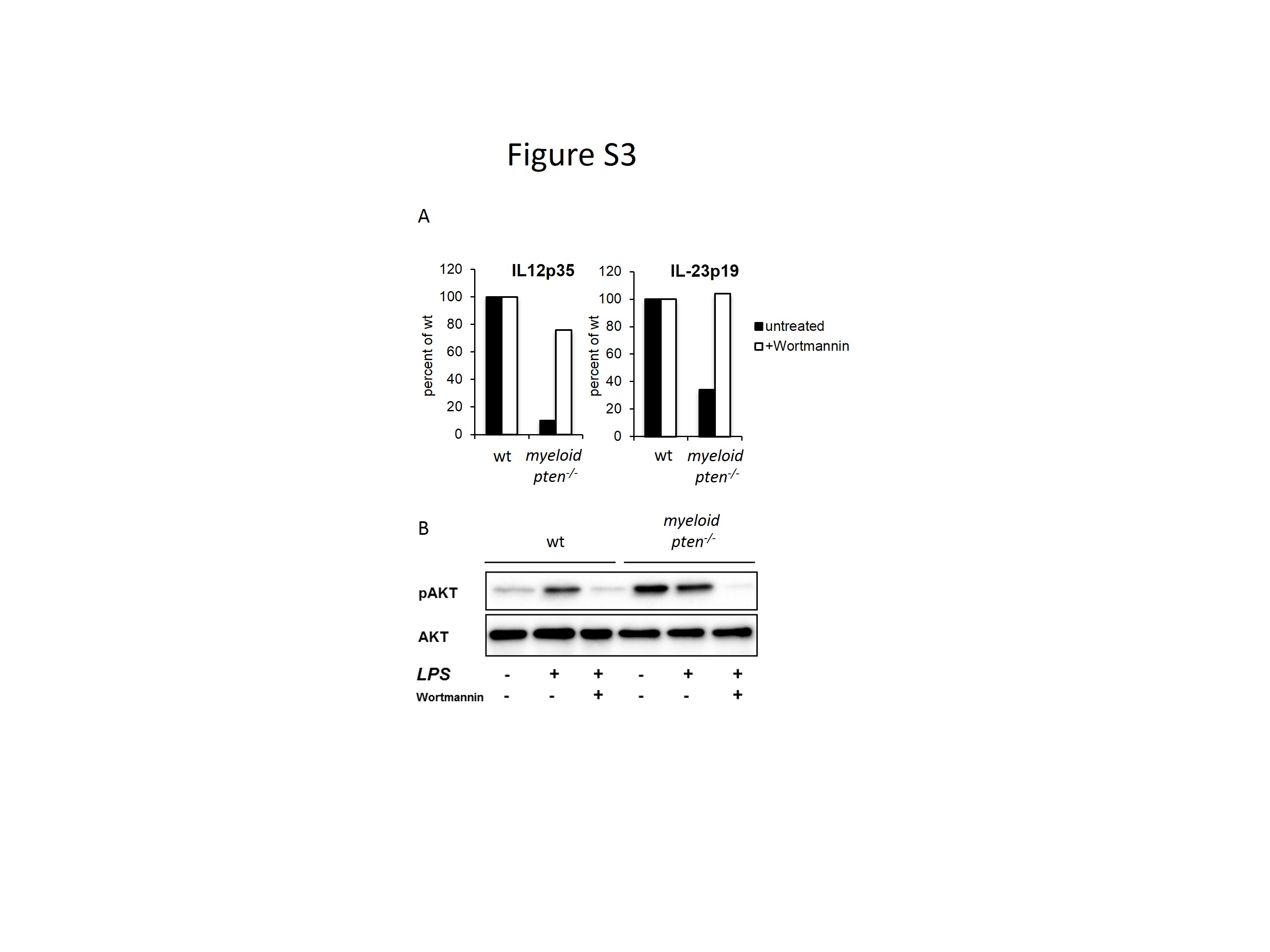

Supplement: Additional file 2: — (A) BMDCs from wt or DC pten -/- animals were analyzed for the expression of the indicated mRNAs by qPCR. (B) BMDCs from wt or DC pten -/- animals were stimulated as indicated and analyzed by western blot for the indicated proteins. (JPEG 214 kb) [file 13075_2015_742_MOESM2_ESM.jpeg]

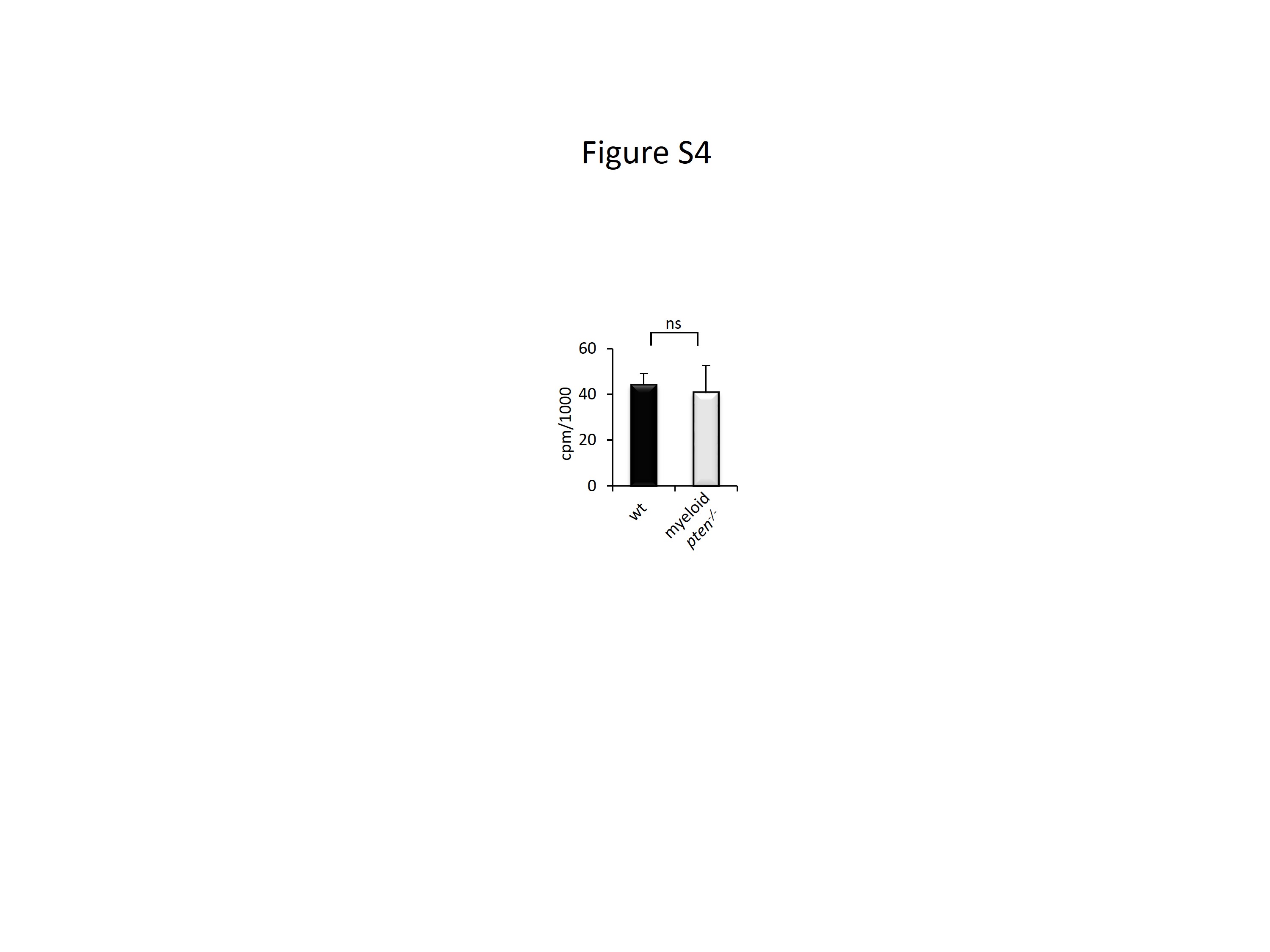

Supplement: Additional file 3: — Cells of the draining LN of wt (n = 4) and myeloid pten -/- (n = 6) mice 2 weeks after induction of CIA were stimulated with plate-bound anti-CD3 for 3 days. H3 Thymidine incorporation was used to quantify proliferation. Data are expressed as means ± s.d. (JPEG 134 kb) [file 13075_2015_742_MOESM3_ESM.jpeg]

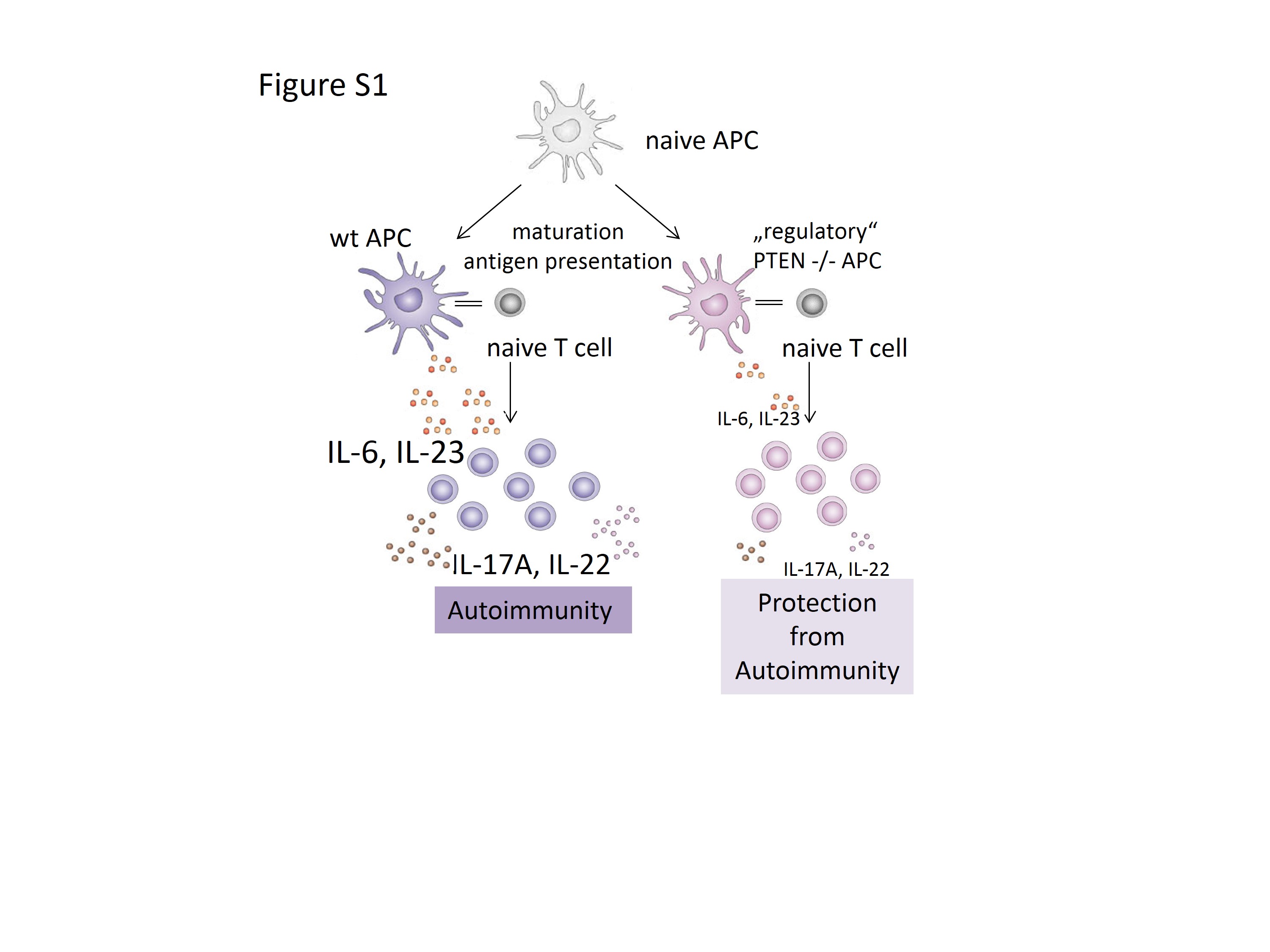

Supplement: Additional file 4: — Schematic representation of APC activation in wt and myeloid pten -/- animals. In wt, APCs are activated and initiate autoimmunity by activating and polarizing T cells toward the Th17 lineage. PTEN-deficient APCs fail to induce autoimmunity due to sustained PI3K signaling, leading to altered signaling and reduced polarizing cytokine expression. (JPEG 294 kb) [file 13075_2015_742_MOESM4_ESM.jpeg]
